# Blood-stage antiplasmodial activity and oocyst formation-blockage of metallo copper-cinchonine complex

**DOI:** 10.3389/fcimb.2022.1047269

**Published:** 2022-12-01

**Authors:** Camila Martins Gomes Morais, Ramayana Morais de Medeiros Brito, Aleksandra Weselucha-Birczyńska, Valeska Santana de Sena Pereira, Jordam William Pereira-Silva, Alexandre Menezes, Felipe Arley Costa Pessoa, Martyna Kucharska, Malwina Birczyńska-Zych, Claudia María Ríos-Velásquez, Valter Ferreira de Andrade-Neto

**Affiliations:** ^1^ Laboratory of Malaria and Toxoplasmosis Biology, Department of Microbiology and Parasitology, Biosciences Center, Federal University of Rio Grande do Norte, Natal, RN, Brazil; ^2^ Post-Graduate Program in Parasitic Biology, Biosciences Center, Federal University of Rio Grande do Norte, Natal, RN, Brazil; ^3^ Laboratory of Immunology and Genomics of Parasites, Department of Parasitology, Institute of Biological Sciences, Federal University of Minas Gerais, Belo Horizonte, MG, Brazil; ^4^ Department of Chemical Physics, Faculty of Chemistry, Jagiellonian University, Kraków, Poland; ^5^ Post-Graduate Program in Biochemistry and Molecular Biology, Biosciences Center, Federal University of Rio Grande do Norte, Natal, RN, Brazil; ^6^ Laboratory of Infectious Disease Ecology in the Amazon, Leônidas and Maria Deane Institute, Fiocruz, Manaus, AM, Brazil; ^7^ Post-Graduate Program in Living Conditions and Health Situations in the Amazon, Leônidas and Maria Deane Institute, Fiocruz, Manaus, AM, Brazil; ^8^ Post-Graduate Program in Biology of Host-Pathogen interaction, Leônidas and Maria Deane Institute, Fiocruz, Manaus, AM, Brazil; ^9^ Department of Infectious and Tropical Diseases, Medical College, Jagiellonian University, Kraków, Poland; ^10^ Department of Infectious Diseases, The University Hospital in Kraków, Kraków, Poland

**Keywords:** *Plasmodium*, malaria treatment, cinchonine, mettalo copper complexes, antimalarial drugs

## Abstract

In the fight against malaria, the key is early treatment with antimalarial chemotherapy, such as artemisinin-based combination treatments (ACTs). However, *Plasmodium* has acquired multidrug resistance, including the emergence of *P. falciparum* strains with resistance to ACT. The development of novel antimalarial molecules, that are capable of interfering in the asexual and sexual blood stages, is important to slow down the transmission in endemic areas. In this work, we studied the ability of the mettalo copper-cinchonine complex to interfere in the sexual and asexual stages of *Plasmodium*. The tested compound in the *in vitro* assay was a cinchonine derivative, named CinCu (Bis[Cinchoninium Tetrachlorocuprate(II)]trihydrate). Its biological functions were assessed by antiplasmodial activity *in vitro* against chloroquine-resistant *P. falciparum* W2 strain. The mice model of *P. berghei* ANKA infection was used to analyze the antimalarial activity of CinCu and chloroquine and their acute toxicity. The oocyst formation-blocking assay was performed by experimental infection of *Anopheles aquasalis* with *P. vivax* infected blood, which was treated with different concentrations of CinCu, cinchonine, and primaquine. We found that CinCu was able to suppress as high as 81.58% of parasitemia *in vitro*, being considered a molecule with high antiplasmodial activity and low toxicity. The *in vivo* analysis showed that CinCu suppressed parasitemia at 34% up to 87.19%, being a partially active molecule against the blood-stage forms of *P. berghei* ANKA, without inducing severe clinical signs in the treated groups. The transmission-blocking assay revealed that both cinchonine and primaquine were able to reduce the infection intensity of *P. vivax* in *A. aquasalis*, leading to a decrease in the number of oocysts recovered from the mosquitoes’ midgut. Regarding the effect of CinCu, the copper-complex was not able to induce inhibition of *P. vivax* infection; however, it was able to induce an important reduction in the intensity of oocyst formation by about 2.4 times. It is plausible that the metallo-compound also be able to interfere with the differentiation of parasite stages and/or ookinete-secreted chitinase into the peritrophic matrix of mosquitoes, promoting a reduction in the number of oocysts formed. Taken together, the results suggest that this compound is promising as a prototype for the development of new antimalarial drugs. Furthermore, our study can draw a new pathway for repositioning already-known antimalarial drugs by editing their chemical structure to improve the antimalarial activity against the asexual and sexual stages of the parasite.

## Introduction

Malaria is a major health problem, especially in developing countries of Africa, Southeast Asia, and South America causing an estimated 241 million cases and 627.000 deaths in 2020 (World Health Organization, 2021). In the Americas, there was an increase in malarial cases due to the growth of transmission in Venezuela, even though Paraguay, El Salvador, and Argentina are now countries with a malaria-free country certificate. In Brazil, 139.023 malaria cases were registered in 2021; during the last decade malaria cases have reduced significantly (from 600.000 to 150.000 cases per year), however it is necessary to find alternative control strategies that support disease elimination (Tableu Public, 2022; World Health Organization, 2021). Five species of *Plasmodium* are capable of infecting humans (*P. falciparum, P. vivax, P. malariae, P. ovale* and *P. knowlesi*), with a recent report of human malaria in Brazil caused by *Plasmodium simium* (Brasil et al., 2017; World Health Organization, 2021). *Plasmodium falciparum* is the most prevalent in Africa and causes severe disease, while *P. vivax* is the most prevalent in other regions, such as South America, and is considered relatively benign. In Brazil, severe malaria had a significant decline, although an increase in *P. vivax* with severe clinical complications was registered (Tableu Public, 2022; Costa et al., 2012; Lacerda et al., 2012; Raposo et al., 2013).

Human malaria is initiated by the bite of infected *Anopheles* mosquitoes, injecting the sporozoites into the skin, which invade blood vessels. For this process to occur, the mosquito is required to ingest gametocytes that are circulating in the bloodstream of the infected host. In the mosquitoes’ midgut, the zygote is assembled and later transformed into a mobile ookinete, which is capable of migrating by peritrophic matrix, crosses the intestinal epithelial cells, and develops into oocysts in the basal lamina; later on, gives rise to sporogony and the presence of the sporozoites into salivary glands (Baton and Ranford-Cartwright, 2005; Angrisano et al., 2012).

Every year, efforts to prevent and control malaria intensify. The key is early treatment with antimalarial chemotherapy, like artemisinin-based combination treatments (ACTs) that have been effective in endemic areas, especially in Southeast Asia (Cui and Su, 2009; Valecha et al., 2010; Ashley et al., 2014; World Health Organization, 2018). However, *Plasmodium* has acquired multidrug resistance to several antimalarial drugs currently available, such as chloroquine (Moore and Lanier, 1961; Payne, 1987), sulfadoxine and pyrimethamine (Hurwitz, 1981), including the emergence of *P. falciparum* strains with resistance to ACT’s in Equatorial Guinea and Southeast Asia (Noedl et al., 2008; Dondorp et al., 2009; Yeung et al., 2009; Ashley et al., 2014; Lu et al., 2017). Most of the current antimalarial drugs are impaired with poor efficacy, high toxicity, and costs; in addition to the resistance occurring faster than the development of new drugs (Gardiner et al., 2009; Garcia-Bustos and Gamo, 2013).

Novel antimalarial molecules have to be capable of interfering in the asexual blood stages, treating the disease symptoms, and decreasing the resistance of parasites. In addition, blocking the transmission of gametocytes from the vertebrate host to the vectors (*Anopheles* mosquitoes) as well as preventing the development of gametes, oocysts, and/or ookinetes in the invertebrate host is important to slow down transmission intensity in endemic areas (Birkholtz et al., 2022). The majority of the antimalarials used nowadays are active only against the early stages of gametocytes but are inactive against the mature stages. Currently, only primaquine is recommended by the WHO and acts in the transmission-blocking of gametocyte mature stages. However, it is not extensively utilized due to its hemolytic toxicity issues in individuals with glucose-6-phosphate-dehydrogenase (G6PD) deficiency (World Health Organization, 2018).

Interest in drugs with therapeutic efficacy already known has grown among researchers, indicating that changes in the structure of these compounds can be useful (Murambiwaa et al., 2011). Based on this principle, several alkaloid molecules with antimalarial potential such as cinchonidine, quinine, cinchonine, and quinidine, derived from plants of the genus *Cinchona* of the Rubiaceae family, have been studied (Hofheinz and Merkli, 1984). These alkaloids belong to a broad group of natural heterocyclic compounds containing nitrogen. Cinchonine is a weak base and crosses the pH gradient of red blood cells to accumulate in the acid vacuoles of the parasites, an important mechanism in its antimalarial action (Weselucha-Birczynska, 2004). Furthermore, the metal coordination with pre-existing antimalarials and bio-organometallic compounds has shown promising efficacy during *in vitro* experiments against chloroquine-sensitive and resistant *P. falciparum* strains (Salas et al., 2013). Copper can be coordinated with several ligands, including cinchonine, and exhibits antiviral activity, treats inflammatory diseases, and microbial infections, and has antimalarial activity (Gokhale et al., 2003; Weselucha-Birczynska, 2004; Mohapatra et al., 2010; Stanila et al., 2011).

Given the interest in the search for new antimalarial treatments with the insertion of metallic compounds, this study aimed to test cinchonine in association with copper (II) to explore the antimalarial activity and its capacity to interfere in asexual and sexual stages in the vertebrate and invertebrate hosts.

## Material and methods

### Studied compound and chemical structure

**Figure 1:**
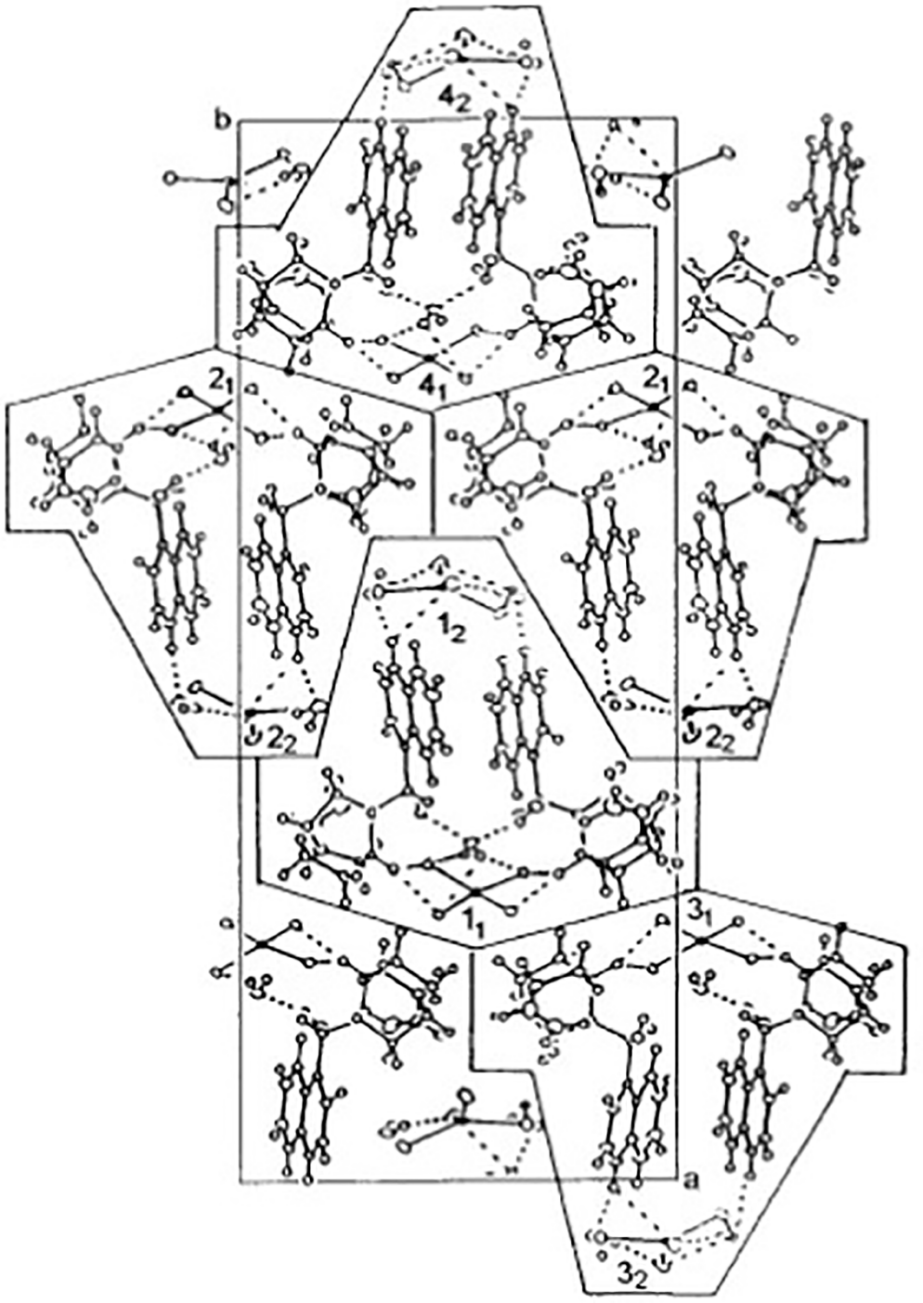
Chemical structures of CuCl_4_-tetrahedra in Bis[Cinchoninium Tetrachlorocuprate(II)]trihydrate (CinCu). Projection of the unit cell along the c-axis at 100 K. The cages contain single molecules [(cinH_2_) ^2+^ (CuCl_4_)^2-^]_2_ ·3H_2_O marked as 1-4 with two geometrically inequivalent (CuCl_4_) ^2-^ anions marked by subscripts 1 and 2. The cages 1-2 and 3-4 form two-dimensional planes parallel to the ac-plane coupled by dispersive forces only. Nitrogens assigned numbers are 1 and 13; oxygen, number 12; carbons, the other numbers smaller than 23; and hydrogen, 23–44 Adapted with permission from {Inorg. Chem. 2001, 40, 18, 4526–4533 Publication Date:July 28, 2001 https://doi.org/10.1021/ic001402a}. Copyright {2001} American Chemical Society.

The compound tested here was a cinchonine derivative synthesized in a previous study (Weselucha-Birczynska et al., 2001) at Jagiellonian University (Krakow, Poland). The asymmetric unit of the compound is formed from two CuCl_4_-tetrahedra in Bis[Cinchoninium Tetrachlorocuprate(II)]trihydrate, here referred to as CinCu (**Figure 1**).

For *in vivo* assays, the compound was solubilized in DMSO (dimethylsulfoxide) and distilled water (1% final concentration). For *in vitro* assays, a stock solution was prepared by dissolving 1 mg of the compound in 1 mL of 1% DMSO.

### *Plasmodium falciparum* maintenance and *in vitro* antimalarial assay

The *in vitro* antiplasmodial activity was performed using a chloroquine-resistant *P. falciparum* W2 strain, according to the modified Trager e Jensen method (Trager and Jensen, 1976; Andradre-Neto et al., 2003). The *P. falciparum* cultures were maintained in O+ human erythrocytes, with 3-5% of hematocrit and 2% parasitemia in RPMI 1640 medium (Sigma-Aldrich) supplemented with 0,5% Albumax I^®^ (Gibco) at 37°C in 5% CO_2_, 5% O_2_ and 90% nitrogen. For culture synchronization, 5% D-Sorbitol (w/v) (Sigma-Aldrich) solution was used consecutively at 48 h intervals, as previously described (Lambros and Vanderberg, 1979).

*Plasmodium falciparum* W2 strain synchronized culture suspension (at 1 to 1.5% parasitemia and 2.5% hematocrit) was seeded in all 96-well culture plates. CinCu stock solution was prepared in freshwater suspension in DMSO at 1% final concentration as an antimalarial drug; the chloroquine stock solution was prepared in distilled water only. For the tests, the stock solutions of CinCu and chloroquine were further diluted in a complete medium (RPMI 1640 supplemented with 0.5% Albumax I^®^). The tested compound CinCu, chloroquine, and 1% DMSO were added to the wells in seven dilutions (1:3 dilution factor). For the seven concentrations of CinCu that were tested, in the lowest concentration of 0.04 ug/mL, the percentage of DMSO was approximately 0.0001% (v/v). Concentrations of 30; 10; 3.33; 1.11; 0.37; 0.12 and 0.04 μg/mL were performed for the CinCu; and concentrations of 2.5; 0.83; 0.27; 0.09; 0.03; 0.01 and 0.003 μg/mL were performed for chloroquine positive control and 1% DMSO for negative control.

The plates were cultured for 48 h at 37°C. Red blood cell smears stained with Giemsa were prepared from each well and observed under an optical microscope (Olympus CX22LED) to determine the IC_50_ (concentration at which 50% inhibition of parasite growth is observed) (Andradre-Neto et al., 2003). Each treatment had two replicates, in independent experimental assays.

### Cytotoxicity assay

The verification of cell viability was assessed by the reduction capacity of the cells on resazurin, the main component of AlamarBlue^®^ as previously described (Fields and Lancaster, 1993). The CinCu and chloroquine were previously diluted with 1% DMSO in culture medium in six concentrations from 200 to 6.25 μg/mL (in 1:2 dilution). For the assay, RAW 264.7 cell line and 2.5× 10^5^ cells *per* well were seeded in 96 wells plates. After 24 h of incubation at 37°C and 5% CO_2_, the culture medium was removed and 100 μL of supplemented medium and 50 μL of the tested compounds were added; for negative control (NC), only supplemented medium was added. The cells were incubated for 24 h in the same conditions mentioned above. After incubation, 15 μL of AlamarBlue^®^ were added to each well; here, wells only with medium were used as blank, and wells with medium plus AlamarBlue^®^ (MAB) were used to calculate the correction factor. The plates were incubated for 4 h and the absorbance was measured at 570 ηm, and 600 ηm. The absorbance values obtained were applied in the equation to calculate the percentage of resazurin reduction (%RR) as follows: {[St570-(St600xCF)/[NC570-(NC600xCF)}x100, where St570 and St600 represent the mean absorbance obtained at 570 and 600 ηm for the samples incubated with the compounds; NC570 and NC600 represent the mean absorbance obtained at 570 and 600 ηm for the negative control. CF represents the correction factor, obtained considering the mean absorbances at 570 and 600 ηm, by the equation: [MAB570 - Blank570]/[MAB600-Blank600]. The data were normalized to express the percentage values, so the negative control values were expressed as 100%.

The test was performed in three replicates and the cytotoxic concentration (CC_50_) values, cell viability, and selectivity index (SI) were calculated. The relative cytotoxicity to antiplasmodial activity for a determined compound was evaluated as a selectivity index (SI), where SI = CC_50_(RAW 264.7 cell line)/IC_50_(*P. falciparum* W2).

### Animals and ethics statement

Healthy Swiss-webster female mice aged 6–8 weeks (25–30 g) were obtained from the central bioterium of the Federal University of Rio Grande do Norte. The mice were fed on a standard pellet diet and water, both given *ad libitum.* They were housed in clean and dry polypropylene cages and maintained with a 12 h light/dark cycle, at 22°C and 30-70% of air humidity, according to the Protocol Organization for Economic Cooperation and Development (OECD/OCDE 425, 2008) (revised OECD, 2022).

All animal tests were approved by the Animal Ethics Committee (CEUA/UFRN) under protocol number 041.048/2017.

### *Plasmodium berghei* ANKA maintenance and antimalarial *in vivo* assay

The *P. berghei* ANKA strain (donated by GHTM – Global Health and Tropical Medicine, unit of the Nova University of Lisbon, Portugal) was maintained *in vivo* in *Swiss* mice by inoculation of 1x10^6^ infected red blood cells in phosphate-buffered saline (PBS) sterile solution by intraperitoneal injection every 7 days in naïve mice. The parasitemia was counted in an optical microscope using blood smears stained with Giemsa (Andrade-Neto, 2000).

To test *in vivo* efficacy of CinCu, the assay was performed as previously described (Bahia et al., 2010; Rios-Velásquez et al., 2013). Mice were randomly divided into three groups of 4 mice/group and received an intraperitoneal injection with 1x10^6^ red blood cells infected with *P. berghei* ANKA. Each group was treated according to the following conditions: (I) control group treated with water/DMSO (vehicle, 1% final volume); (II) treated with Bis[Cinchoninium Tetrachlorocuprate(II)]trihydrate (CinCu); (III) treated with chloroquine; Group II and III were treated at doses of 10 mg/kg, 20 mg/kg, 30 mg/kg and 60 mg/kg. The infected mice were treated daily by oral route (*per gavage*) for 4 consecutive days, starting at day 0 after infection. On the fifth and seventh days after infection, blood smears were prepared from the tail of each animal to determine the parasitemia and its suppression percentage as described by Krettli et al. (2001). Additionally, each mouse was observed daily to determine survival time. All tests were performed in three independent experiments.

### Acute toxicity

Acute toxicity was performed according to the revised guidelines OECD (2022). *Swiss* female mice were randomly divided into groups containing 3 animals/group in the same conditions of the antimalarial *in vivo* assay. Each mouse, from the II and III groups, was treated *per gavage* with a single dose of 300 mg/kg body weight. The animals were monitored daily for 14 days for signs of illness such as piloerection, diarrhea, salivation, changes in the eyes and mucous membranes, behavior patterns, and somatomotor activity with special attention to tremors, convulsions, salivation, lethargy, sleep, coma, and weight loss. On day 14 or in case of suffering, the animals were euthanized with anesthesia lethal dose (xylazine/ketamine).

### Transmission blocking of *Plasmodium vivax* in *Anopheles aquasalis* assay

Experiments were performed using *Anopheles aquasalis* adult females from the colony of the Laboratory of Infectious Disease Ecology in Amazon facility from Leônidas and Maria Deane Institute (ILMD-Fiocruz AM). Adult female mosquitoes were reared in cages kept in insectary conditions at 27°C and 80% relative humidity on a 12 h light/dark cycle and fed on 10% sucrose solution (Rios-Velásquez et al., 2013). For the experiments, it was used blood from adult patients over 18 years old, who agreed to participate in the project as volunteers and signed informed consent documents. The patients were infected with two crosses of parasitemia (501 – 10.000 parasites/μL) of *P. vivax*; the infection was diagnosed by thick blood smear method at the Dr. Heitor Vieira Dourado Tropical Medicine Foundation (FMT-HVD). For each patient, 3 mL of blood were collected in heparinized Vacutainer^®^ tubes. This procedure was approved by the Ethical Review Committee at FMT-HVD (CAAE 39706514.2.00000.0005).

Adult mosquito females, 3 – 5 days old, were sugar starved overnight prior to experimental infection *via* membrane feeding assay, as described by Rios-Velásquez et al. (2013) (Rios-Velásquez et al., 2013). Mosquitoes were separated into seven experimental groups, each one with 100 individuals, treated with different doses of cinchonine (Cin 1 µg/mL and 0.23 µg/mL); Copper-coordinated cinchonine (CinCu 0.78 µg/mL and 0.06 µg/mL); and primaquine (PQ 1 µg/mL and 10 µg/mL), all diluted in 1% DMSO. Untreated infected blood was used as control. Every assay was realized in biological triplicate.

After the infective blood meal, only fully engorged mosquitoes were transferred to rearing containers, fed with 10% sucrose *ad libitum*, and maintained in the insectary for the development of the parasite infection. On the seventh day after the infective blood meal, the midgut of the mosquitoes was dissected in phosphate-buffered saline, stained with 2% commercial Mercurochrome (Merbromin), covered with a coverglass, and examined for the presence of oocysts under an optical microscope at 20x magnification. The number of oocysts was quantified in every midgut dissected.

### Statistical analysis

The differences between treated and control groups of the *in vitro* assays were analyzed with a two-tailed Student’s *t*-test or Simple Analysis of Variance (ANOVA) followed by Tukey test; with a significance level of 5%.

The inhibitory concentrations in antimalarial *in vitro* and cytotoxicity assay, IC_50_ values were determined by non-linear regression with dose-response curves categorized into high activity (IC_50_ ≤ 10 μg/ml); moderate activity (10< IC_50_< 100 μg/ml); low activity (IC_50_ > 100 μg/ml) (Meneguetti et al., 2014).

The infection rate of the mosquitoes in the transmission-blocking assay for each treatment was calculated by the number of individuals who had at least one oocyst divided by the number of dissected mosquitoes and multiplied by 100. Oocyst infection intensity was calculated by dividing the total number of oocysts by the total number of infected mosquitoes. The inhibition percentage of each compound was calculated as previously described (Fabbri et al., 2021). To evaluate the differences in the oocyst number among the groups was used Kurskal-Wallis test with Dunn’s multiple comparison test. All statistical tests were performed with GraphPad Prism 6.0.

## Results

### Antiplasmodial and cytotoxicity *in vitro* assays

**Figure 2:**
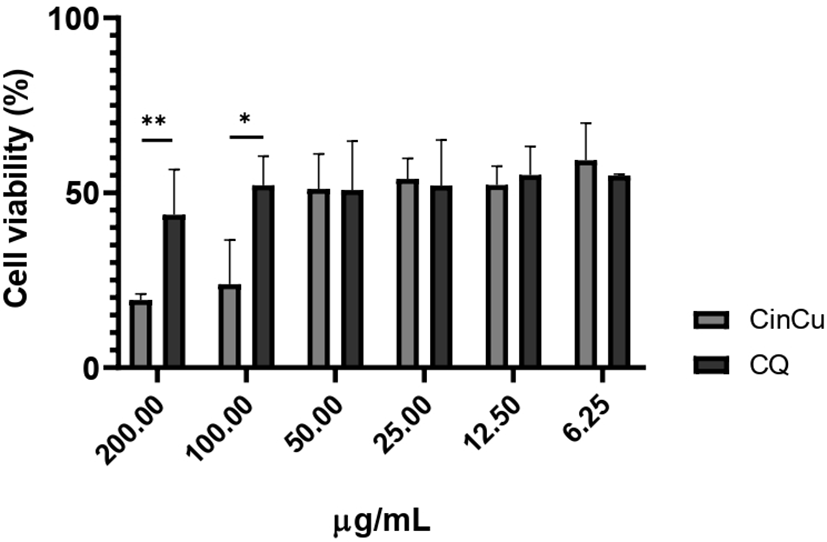
*In vitro* cytotoxicity analysis of CinCu and chloroquine in RAW264.7 cell line. CQ: chloroquine; CinCu: Copper-coordinated cinchonine. Results are presented as mean ± SD of three replicates. The data displayed here represents the concentrations of compounds that guarantee at least 50% cell viability (CC50). ** p<0.01; * p<0.05.

**Figure 3:**
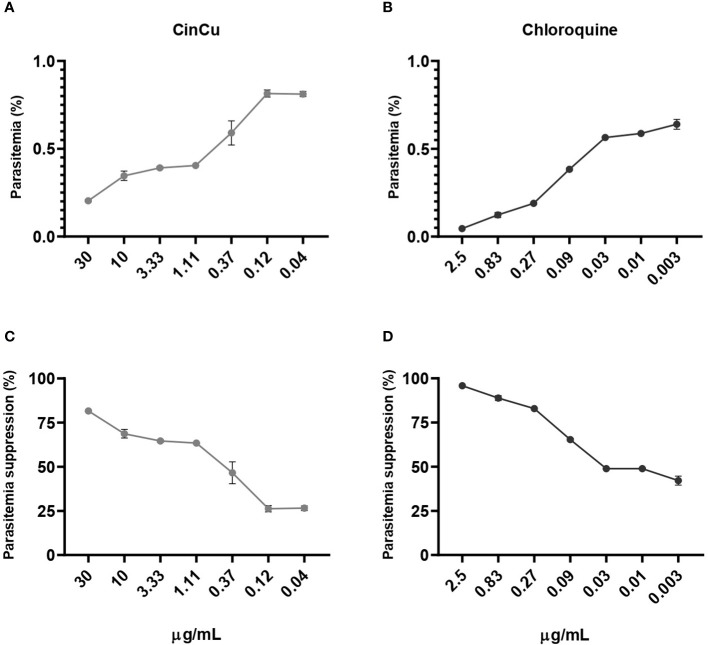
*In vitro* antiplasmodial activity of the CinCu and chloroquine against *P*. *falciparum* W2 strain. The dose-dependent curve of the parasitemia of CinCu **(A)** and chloroquine **(B)**. The parasitemia suppression of CinCu **(C)** and chloroquine **(D)**, respectively. Results are presented as mean ± SD of two replicates in two independent experiments. Suppression of the parasite growth was calculated in relation to control cultures with no drugs.

The cytotoxicity assay revealed that both CinCu and chloroquine were able to maintain at least 50% of cell viability in four out of the six tested concentrations (**Figure 2**). The evaluation of CinCu antiplasmodial activity over a chloroquine-resistant *P. falciparum* W2 strain revealed that this molecule is able to induce a significant parasitemia suppression, as high as 81.58% of suppression in the highest concentration tested (30 μg/mL), and maintaining a suppression higher than 60% for the concentrations of 10 μg/mL, 3.33 μg/mL and 1.11 μg/mL (**Figures 3A, C**). In addition, the chloroquine treatment revealed a significant parasitemia suppression for the three first tested concentrations (2.5, 0.83, and 0.27 μg/mL), ranging from 95.8% to 82.8% (**Figures 3B, D**). According to the IC_50_ values, the CinCu can be considered a molecule with high antiplasmodial activity (10< IC_50_< 100 μg/mL) and low toxicity (SI=155.57 μg/mL) (**Table 1**).

**Table 1:** *In vitro* analysis of antiplasmodial and cytotoxicity activity of Bis[Cinchoninium Tetrachlorocuprate(II)]trihydrate (CinCu) and chloroquine.

	${\text{CC}}_{50}^{\text{a}}$		${\text{IC}}_{50}^{\text{b}}$		SI^c^
	Mean (μg/mL)	SD	Mean (μg/mL)	SD	
**Chloroquine**	117.3	4.15	0.12	0.01	994.16
**CinCu**	71.58	17.1	0.46	0.13	155.57

a CC_50_ mean values for RAW264.7 cell line treated with different concentrations of CinCu and chloroquine.

b *Plasmodium falciparum* W2 IC_50_: High activity (IC_50_ ≤ 10 μg/mL); moderate activity (10< IC_50_< 100 μg/mL); low activity (IC_50_ > 100 μg/mL).

c SI values >10 indicate low toxicity; values<10 indicate moderate to the high toxicity of the compounds.

Values are represented as the mean of three replicates ± standard error of the mean at 95% confidence intervals with lower and upper limits. SD: Standard deviation.

### *In vivo* antimalarial activity

**Figure 4:**
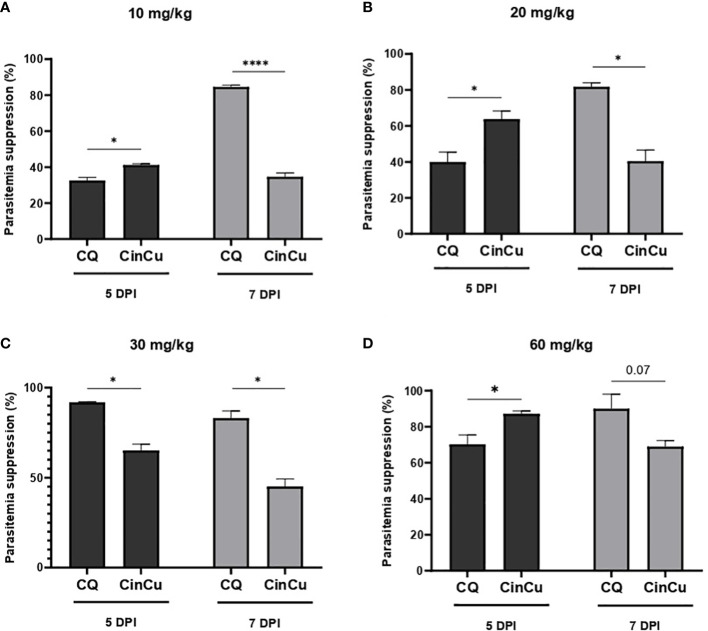
*In vivo* analysis of the CinCu and chloroquine effect on parasitemia suppression of *P. berghei* ANKA infection. The parasitemia suppression activity was evaluated on the fifth and seventh days post-infection, after treatment with 10 mg/Kg **(A)**, 20 mg/Kg **(B)**, 30 mg/Kg **(C)**, and 60 mg/Kg **(D)** of CinCu and chloroquine. Results are presented as mean ± SD of three independent experiments. * p<0.05; **** p<0.0001.

The results obtained showed significant parasitemia suppression of CinCu (superior to 30%) in all doses administered, being considered a partially active molecule. On the 5^th^ day, CinCu displayed higher parasitemia suppression when compared to chloroquine for the doses of 10, 20, and 60 mg/kg (**Figures 4A, B, D**). For the dose of 30 mg/Kg, CinCu was able to maintain a considerable parasitemia suppression, although lower than chloroquine (**Figure 4C**); the suppression activity of CinCu ranged from 34.69% on the 7^th^ day for the lower dose (10 mg/Kg) to 87.19% on the 5^th^ for the highest dose (60 mg/Kg) (**Table 2**). The chloroquine showed a parasitemia suppression ranging from 26.36% on the 5^th^ day to 84.59% on the 7^th^ day in the lower dose (10 mg/Kg) to 70.24% on the 5^th^ day to 90% on the 7^th^ day for the highest dose (60 mg/Kg) (**Table 2**).

**Table 2:** Antimalarial activity of CinCu and chloroquine in mice infected with *Plasmodium berghei* ANKA.

Compounds or reference drug	Dose (mg/kg) orally 4x	% Parasitemia suppression (SD)^a^		Half-survival time in days	Antimalarial activity^b^
		5^th^	7^th^		
**CinCu**	60	87.19 (1.55)	69 (3.29)	16	Active
	0^c^			10
	30	65.16 (4.92)	45.10 (5.91)	11	Partial^d^
	0^c^			9
	20	63.84 (6.3)	40.44 (8.75)	25	Partial^d^
	0^c^			10
	10	41.2 (1.12)	34.69 (3.61)	14	Partial
	0^c^			19
**Chloroquine**	60	70.24 (5.25)	90 (8.08)	25	Active
	0^c^			10
	30	91.86 (0.44)	83.1 (5.59)	25	Active
	0^c^			9
	20	39.96 (7.71)	81.73 (3.15)	25	Active^d^
	0^c^			10
	10	26.36 (10.9)	84.59 (1.66)	25	Active^d^
	0^c^			19

a Reduction of parasitemia compared to the non-treated control mice. Values are expressed as mean ( ± SD).

b Compounds that reduce ≥30% parasitemia are considered partially active.

c Control non-treated group (vehicle: water/DMSO 1%).

d Only on the 7th day.

Parasitemia suppression was calculated relating to the group of untreated control mice. The values are represented as the mean of three values ± standard deviation of 5° and 7° days after infection.

**Figure 5:**
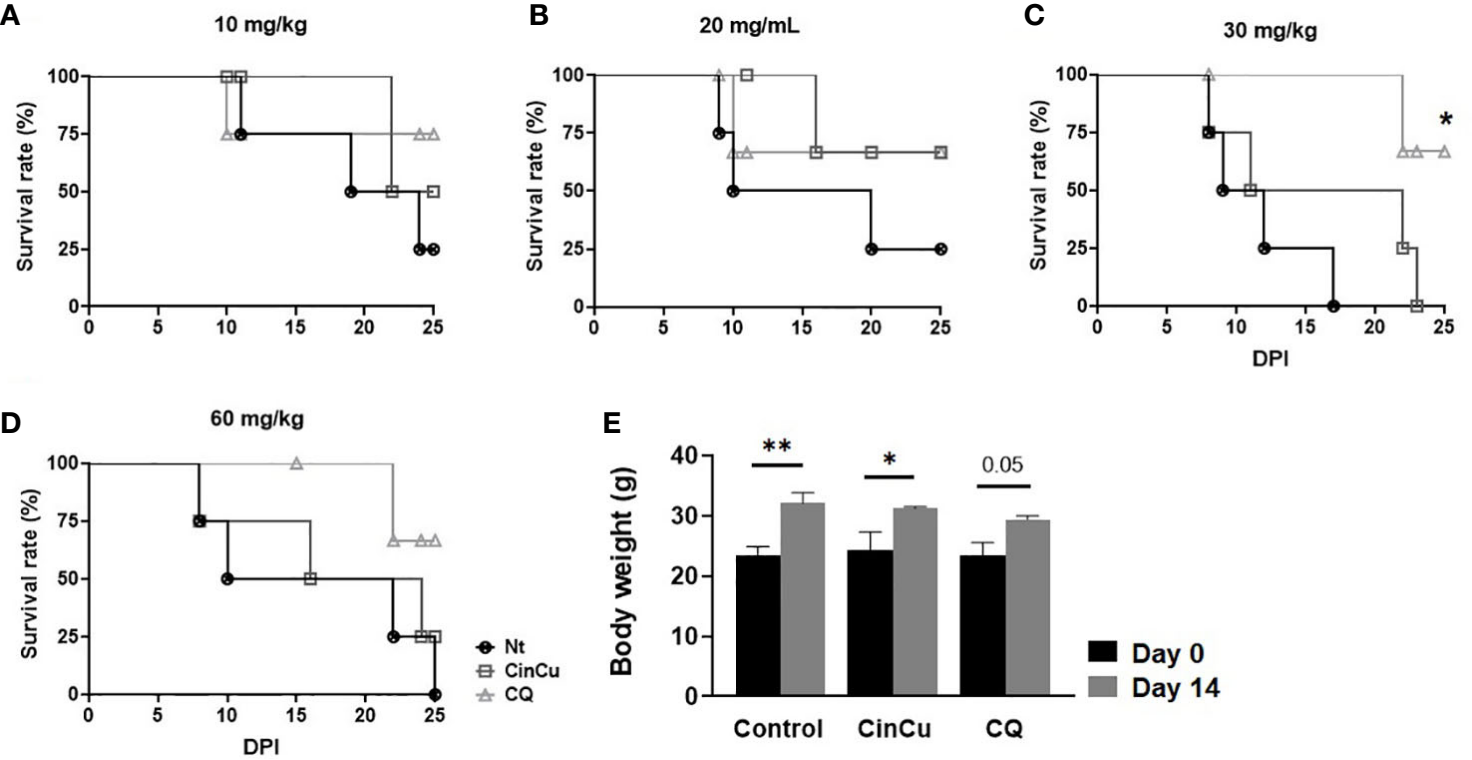
The survival rate of mice infected with *P. berghei* ANKA and treated with CinCu or Chloroquine. The survival rates of mice treated with 10 mg/Kg **(A)**, 20 mg/Kg **(B)**, 30 mg/Kg **(C)**, and 60 mg/kg **(D)** were determined and analyzed by the Kaplan-Meier method with Log-rank (Mantel-Cox) test. **(E)** Body weight of mice before (day 0) and after (day 14) treatment with CinCu or CQ at 300 mg/Kg in the acute toxicity assay. Nt: non-treated group (control); CinCu: mice treated with CinCu; CQ: mice treated with chloroquine; DPI: days post-infection. **p<0.01; *p<0.05.

**Figures 5A**–**D** show the survival rate of mice infected with *P. berghei* Anka and treated with CinCu or chloroquine. Mice treated with CinCu in doses of 20 and 60 mg/Kg reached 50% mortality later than the untreated control group (**Figures 5B, C**, **Table 2**).

### Acute toxicity *in vivo*

Mice treated with a 300 mg/Kg acute dose of CinCu and chloroquine were monitored for 14 days, and the clinical signs observed in the animals are displayed in **Table 3**. The only alteration observed was piloerection on the 4th day after treatment for both CinCu and chloroquine. At the end of the 14th day, all animals were alive and no weight loss was registered (**Figure 5E**).

**Table 3:** Toxicity signals after treatment with CinCu and chloroquine (CQ) at 300 mg/kg dose.

Parameters observed	Toxicity signals (at 300 mg/kg)	
	CinCu	CQ
Skin/Fur	A	A
Eyes and Mucous Membranes	N.a	N.a
Cardiac and respiratory signs	N.a	N.a
Behavior pattern	N.a	N.a
Somatomotor activity	N.a	N.a
Salivation	N.a	N.a
Tremors	N.a	N.a
Convulsions	N.a	N.a
Lethargy	N.a	N.a
Sleep	N.a	N.a
Coma	N.a	N.a
Mortality	N.a	N.a

N.a.: No alterations; A.: Alteration.

### Effects of CinCu in *P. vivax* transmission-blocking and oocyst formation in *Anopheles aquasalis*

In these experiments primaquine was used as a control because of its use as antimalarial medicine. The infection rate for *P. vivax* in *A. aquasalis* was high for all the compounds evaluated, except for primaquine 1 μg/mL and 10 μg/mL, and cinchonine 1 μg/mL; however, the difference was not significant among them (**Table 4**).

**Table 4:** Effect of blood treatment with cinchonine, primaquine, and CinCu on *P. vivax* infection rate, oocyst intensity and oocyst inhibition in *A. aquasalis*.

	Tested concentration (μg/mL)	Total engorged/dissected mosquitoes	Rate of infection (%)	% Rate of infection Inhibition	Oocyst infection intensity*
**Control**	0	198/189	89.02	NA	107.04
**Primaquine**	1	211/204	28.83	67.62	21.53
	10	215/187	54.55	38.73	39
**Cinchonine**	0.23	219/191	90.59	0	27.35
	1	207/178	20	77.53	14.83
**CinCu**	0.06	232/189	97.22	0	50.3
	0.78	175/159	88.46	0.63	43.78

Every experiment was performed in biological triplicate.

NA: Not applicable.

* Mean values for oocysts.

**Figure 6:**
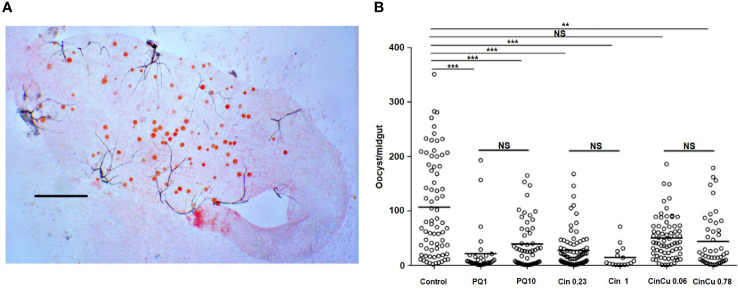
Oocyst infection intensity of *P. vivax* in *A*. *aquasalis* after feeding on blood treated with cinchonine, primaquine, and CinCu. **(A)** Representative image of *P. vivax* oocysts in the midgut of *A*. *aquasalis;* the slide was stained with 2% commercial Mercurochrome (Merbromin); scale bar: 250 µm. **(B)** Number of oocysts *per* midgut of *A*. *aquasalis* after blood meal. PQ 1: primaquine 1 µg/mL; PQ 10: primaquine 10 µg/mL; Cin 0.23: cinchonine 0.23 µg/mL; Cin 1: cinchonine 1 µg/mL; Cin+Cu 0.06: cinchonine in association with copper at 0.06 µg/mL; Cin+Cu 0,78: cinchonine in association with copper at 0.78 µg/mL NS: non-significant, ** p<0.01, *** p< 0.001.

The infection intensity, evaluated by the mean of oocysts per midgut (**Figure 6A**), was higher in the control and significantly lower in both concentrations used for primaquine and cinchonine, and CinCu at 0.78 μg/mL (**Table 4**, **Figure 6B**). These results showed that blood treatment with the evaluated concentrations of primaquine and cinchonine significantly reduced the oocyst formation. Primaquine (1 μg/mL and 10 μg/mL), and cinchonine (1 μg/mL) showed a high percentage of inhibition of the infection rates for *P. vivax*. The previous blood treatment with CinCu did not affect the inhibition of *P. vivax*, as observed in the mosquito’s infection rate; however, it was able to reduce the oocyst intensity (**Table 4**).

## Discussion

Due to widespread resistance to current antimalarial drugs, there is an urgent need for research on effective, safe, and affordable antimalarial compounds, since the main tool for malaria control is chemotherapy (Na-Bangchang and Karbwang, 2013; Fabbri et al., 2021). Furthermore, a drug must have both anti-recrudescence and blood schizontocidal activity, besides acting in gametocytes stages and/or sporogonic forms with minimal side effects.

Modifications of the alkaline skeleton of *Cinchona* derivatives have been one of the most successful strategies in the development of antimalarial drugs (Dinio et al., 2012). There are about 35 different alkaloids in the bark of the *Cinchona* tree, of which four: cinchonidine, quinine, cinchonine, and quinidine, occur in the largest amount, and differ in the substituents on the C19 carbon atom and the absolute configuration on the C8 and C9 stereogenic centers. Previous *in vivo* tests showed cinchonine as a compound with activity higher than quinine and cinchonidine. Tests performed on *P. gallinaceum* and *P. berghei*, showed that cinchonine and quinidine exhibited greater compatibility with the site of the high affinity of parasite uptake, ferriprotoporphyrin (Warhurst, 1981). Cinchonine is a weak base that crosses the pH gradient of red blood cells to accumulate in the acid vacuoles of the *Plasmodium* parasites and is selectively trapped inside the food vacuole in an ion-trapping mechanism (Weselucha-Birczynska, 2004).

To reduce the toxicity of knowing antimalarial drugs, promising approaches involving the use of transition metal complexes in chemotherapeutic agents are being studied (Warhurst, 1981; Sekhon and Bimal, 2012). Metal complexes have been used for a variety of diseases, including anti-parasitic agents, showing selectivity to parasitic cells (Orvig and Abrams, 1999; Crans and Meade, 2013). In this sense, the antimalarial properties of various metal complexes are being currently evaluated. The coordination of a pharmacological system with metals has shown results of superior activity of biodistribution, absorption, metabolism, minimized side effects, extending the time of drug in the organism, and reaching the biological target more precisely (Sanchez-Delgado and Anzellotti, 2004; Navarro et al., 2010).

Several copper complexes produce considerable antimalarial activity (Bahl et al., 2010; Weintraub et al., 2015; Medici et al., 2015). The use of Cu(II)/Cu(I) in antimalarial activity probably occurs due to oxidation-reduction reactions that interrupt mitochondrial respiration inducing reactive oxygen species (ROS) (Navarro et al., 2010). In our study, CinCu was able to suppress above 50% of parasitemia *in vitro* against the chloroquine-resistant *P. falciparum* W2 strain, in the first four concentrations tested. The non-protonated form of copper can pass through the vacuolar membrane, where it changes to a protonated form and becomes trapped (Olliaro, 2001). Copper homeostasis is necessary for the asexual phase for the growth of *P. falciparum*, therefore its disturbance by introducing additional copper ions may improve the antimalarial effect of CinCu (Asahi et al., 2016). This mode of action, in combination with cinchonine, acts on the food vacuole of *Plasmodium* enhancing the antimalarial activity. Studies showing the antiplasmodial activity of copper-coordinated complexes have been reported in the past years (Gokhale et al., 2006; Tapanelli et al., 2017). The analysis of copper(II) nanohybrid solids revealed the capacity of these compounds to induce parasitemia suppression of *P. falciparum in vitro*, an effect probably due to plasmepsin II inhibition (Mohapatra et al., 2010). Analysis of copper(II) complexes of pyridine-2-carboxamidrazones also showed efficient activity against *P. falciparum in vitro* (Gokhale et al., 2003). Based on these studies, it is plausible to assume the effect of copper-coordination on the enhancement of antimalarial activity seen in many compounds.

Experimental analysis of Fe(III), Cu(II), and Zn(II) complexes with quinine and mefloquine showed similar antimalarial activity *in vivo*, but expressive toxicity of the ligands or complexes in terms of the alkaline phosphatase activity level (Obaleye et al., 2009). A previous study showed the ability of two hexahydroquinolines complexes to reduce the parasitemia *in vivo* by 22% and 43% at 70 mg/Kg dose; and 65% and 91% at 100 mg/kg (Vanaerschot et al., 2017).

Here, we found that CinCu presented an antimalarial activity during experimental *P. berghei* murine infection being active at all doses tested; at the lowest dose, it was able to inhibit parasitemia in a range of 34-41%, while at the highest doses tested the suppression ranged from 40-87%, about twice as effective. These results are very consistent and promising for new prototypes of antimalarials. The approach used in this study, from the optimization of current drug regimens and/or modifications in old antimalarial agents, opens up interesting perspectives for the development of new compounds that can be used in chemotherapeutic combinations; as well as the discovery of new targets (Belete, 2020).

Transmission-blocking activity is an important result for drugs that also act in asexual blood phases of *Plasmodium*, being able to reduce or even interrupt the parasite cycle in the two hosts: the vertebrate and invertebrate (Wadi et al., 2019). The use of artesunate, quinine, and primaquine for transmission-blocking assays has been reported (Chotivanich et al., 2006), and primaquine is the only drug with transmission-blocking activity recommended by the WHO; however, its use is limited due to toxicity in individuals with G6PD deficiency (White et al., 2012; Wadi et al., 2019). In this sense, the intensification of the search for new compounds that display the ability to interfere with the parasite’s sexual stage is essential to block the transmission cycle. Based on the antiplasmodial activities, *in vivo* and *in vitro*, displayed here by CinCu, we tested the effect of this cinchonine-copper complex on the transmission of *P. vivax* to its vector, *A. aquasalis*. Results obtained by membrane feeding assays showed that primaquine and cinchonine are able to limit the infection rate in *A. aquasalis* infected with *P. vivax.* Despite the variability in the parasite infection rates, their reduction was expected because primaquine and cinchonine are used as antimalarial treatments. One important result is that all the studied compounds limited the oocyst infection intensity.

In a study performed by Vanaerschot et al. (2017), two hexahydroquinolines complexes were shown to reduce 97% and 57% of mosquito infection, whilst oocyst density *per* midgut was reduced by 21% in *A. stephensi* model (Vanaerschot et al., 2017). The presence of copper in antimalarials has shown a potent effect against asexual blood forms in *in vitro* tests with *P. falciparum* (Tapanelli et al., 2017). In addition, the antimalarial activity for CinCu complex might be associated with the four coordinated copper planar geometry that has been suggested to promote an easier internalization of the drug (Subczynski et al., 1987). Blood treatment with CinCu caused an important reduction in the infection intensity, indicating that higher concentrations could be used as transmission blockers. However, the analysis of the higher concentrations effect displayed here by the compounds will require the evaluation of the insecticide effect and, also, the effect on the mosquito salivary glands invasion.

Metal coordination of drugs represents an improvement in the research of new antimalarial drugs. The redox activity of copper ions along with the biogenicity, the stability of copper complex compounds in the bloodstream, the ability to penetrate the cell membrane and fluids much easier than the organic ligands, and the highly promising therapeutic results *in vitro* and *in vivo* prove the potential to become widely used in clinical practice (Salas et al., 2013). This approach allows the use of low doses of the drug considered toxic, reducing the risks to the patient and improving the drug efficiency on the target, decreasing the risk of developing resistance (Baird, 2005) as well as improving the selectivity index or making the metal more inert during its interaction with biomolecules (Krungkrai and Yutharvong, 1987; Sanchez-Delgado and Anzellotti, 2004).

The work reported here shows that the copper-cinchonine complex displays significant antimalarial activity when tested against the chloroquine-resistant *Plasmodium falciparum* strain with low *in vitro* cytotoxicity, followed by a satisfactory suppression of parasitemia *in vivo.* The same promising results are shown by CinCu displaying moderate activity over the oocyst intensity in *A. aquasalis*. Further research on the physical-chemical characteristics of copper metal complexes, in addition to improved methods of associating these metals with compounds, and their mechanisms of antiparasitic action need to be carried out, including research on the potential of these metallo-complexes in inhibiting the transmission of gametocytic forms to vector mosquitoes.

## Data availability statement

The original contributions presented in the study are included in the article/supplementary material. Further inquiries can be directed to the corresponding authors.

## Ethics statement

The procedure was approved by the Ethical Review Committee at FMT-HVD (CAAE 39706514.2.00000.0005). The patients/participants provided their written informed consent to participate in this study. All animal tests were approved by Animal Ethics Committee (CEUA/UFRN) under protocol number 041.048/2017.

## Author contributions

CMGM, CMR-V, and VFA-N designed the study; CMGM and RMMB performed the experiments; AW-B was responsible for the chemical study and synthesis of the chemical compound; MK and MB-Z contributed to chemical studies and experiments; VSSP and RMMB were responsible for the maintenance of *Plasmodium falciparum in vitro* culture and contributed to the tests; JWP-S, AM, FACP, and CMR-V contributed to *P. vivax* transmission-blocking assays; CM and VFA-N wrote the first draft of the manuscript; CMGM, RMMB, AW-B, FACP, CMR-V, and VFA-N analyzed the data; CMMG, RMMB, CMR-V, AW-B, and VFA-N wrote the manuscript; VFA-N and CMR-V coordinated the study. All authors contributed to manuscript revision, read, and approved the submitted version.

## Funding

The authors also would like to thank the Brazilian Research Support Agencies (CNPq, CAPES, FAPEAM, and PROEP ILMD Fiocruz Program) and AWB (subsidy from the Faculty of Chemistry of the Jagiellonian University in Krakó w, Poland). RMMB is the recipient of the Ph.D. scholarship and also would like to thank CAPES; VFA-N (Process # 306036/2019-3) and FACP (Process # 311726/2021-6) are thankful for the CNPq Research Productivity Fellowship.

## Acknowledgments

The authors would like to thank Silvany G. Brito (Biological Sciences-UFRN/Scientific Initiation Student), Alison Michel S. de Oliveira, Sarah R. D. Batista, Karen M. Pereira (Biomedicine-UFRN/Scientific Initiation Students) for their support and logistics. 

## Conflict of interest

The authors declare that the research was conducted in the absence of any commercial or financial relationships that could be construed as a potential conflict of interest.

## Publisher’s note

All claims expressed in this article are solely those of the authors and do not necessarily represent those of their affiliated organizations, or those of the publisher, the editors and the reviewers. Any product that may be evaluated in this article, or claim that may be made by its manufacturer, is not guaranteed or endorsed by the publisher.

## References

[B1] Andrade-Neto V. F. (2000). Atividade antimalárica de extratos brutos, frações semi-purificadas e de compostos quimicamente definidos isolados de plantas utilizadas Na medicina popular (Belo Horizonte (MG: Federal University of Minas Gerais).

[B2] Andradre-Neto V. F. Brandão M. G. L. Stehmann J. R. Oliveira L. A. Krettli A. U. (2003). Antimalarial activity of *Cinchona*-like plants udes to treat fever and malária in Brazil. J. Ethnopharmacol. 87 (2-3), 253–256. doi: 10.1016/s0378-8741(03)00141-7 12860318

[B3] Angrisano F. Tan Y. H. Sturm A. McFadden G. I. Baum J. (2012). Malaria parasite colonisation of the mosquito midgut – placing the *Plasmodium* ookinete centre stage. Int. J. Parasitol. 42 (6), 519–527. doi: 10.1016/j.ijpara.2012.02.004 22406332

[B4] Asahi H. Kobayashi F. Inoue S. I. Niikura M. Yagita K. Tolba M. E. (2016). Copper homeostasis for the developmental progression of intraerythrocytic malarial parasite. Curr. Top. Med. Chem. 16 (27), 3048–3057. doi: 10.2174/1568026616999160215151704 26881705 PMC5068492

[B5] Ashley E. A. Dhorda M. Fairhurst R. M. Amaratunga C. Parath L. Suon S. . (2014). Spread of artemisinin resistance in *Plasmodium falciparum* malaria. N Engl. J. Med. 371, 411–423. doi: 10.1056/NEJMoa1314981 25075834 PMC4143591

[B6] Tableu Public (2022) (Malária – Brasil: Ministério da Saúde). Available at: https://public.tableau.com/app/profile/mal.ria.brasil (Accessed September 12, 2022).

[B7] Bahia A. C. Kubota M. S. Tempone A. J. Pinheiro W. D. Tadei W. P. Secundino N. F. C. . (2010). *Anopheles aquasalis* infected by *Plasmodium vivax* displays unique gene expression profiles when compared to other malaria vectors and plasmodia. PloS One 5 (3), e9795. doi: 10.1371/journal.pone.0009795 20339545 PMC2842430

[B8] Bahl D. Athar F. Soares M. B. P. Santos De Sa M. Moreira D. R. M. Srivastava R. M. . (2010). Structure-activity relationships of mononuclear metal-thiosemicarbazone complexes endowed with potent antiplasmodial and antiamoebic activities. Bioorg. Med. Chem. 18 (18), 6857–6864. doi: 10.1016/j.bmc.2010.07.039 20719524

[B9] Baird J. K. (2005). Effectiveness of antimalarials drugs. N Engl. J. Med. 352 (15), 1565–1577. doi: 10.1056/NEJMra043207 15829537

[B10] Baton L. A. Ranford-Cartwright L. C. (2005). Spreading the seeds of million-murdering death: metamorphoses of malaria in the mosquito. Trends Parasitol. 21 (12), 573–580. doi: 10.1016/j.pt.2005.09.012 16236552

[B11] Belete T. M. (2020). Recent progress in the development of new antimalarial drugs with novel targets. Drug Des. Devel. Ther. 14, 3875–3889. doi: 10.2147/DDDT.S265602 PMC7519860 33061294

[B12] Birkholtz L. M. Alano P. Leroy D. (2022). Transmission-blocking drugs for malaria elimination. Trends Parasitol. 38 (5), 390–403. doi: 10.1016/j.pt.2022.01.011 35190283

[B13] Brasil P. Zalis M. G. Pina-Costa A. Siqueira A. M. Bianco-Junior C. Silva S. . (2017). Outbreak of human malaria caused by *Plasmodium simium* in the Atlantic forest in Rio de Janeiro: a molecular epidemiological investigation. Lancet Glob. Health 5 (10), e1038–e10465. doi: 10.1016/S2214-109X(17)30333-9 28867401

[B14] Chotivanich K. Sattabongkot J. Udomsangpetch R. Looareesuwan S. Day N. P. J. Coleman R. E. . (2006). Transmission-blocking activities of quinine, primaquine, and artesunate. Antimacrob. Agents Chemother. 50 (6), 1927–1930. doi: 10.1125/AAC.01472-05 PMC1479118 16723547

[B15] Costa F. T. Lopes S. C. Albrecht L. Ataíde R. Siqueira A. M. Souza R. M. . (2012). On the pathogenesis of *Plasmodium vivax* malaria: perspectives from the Brazilian field. Int. J. Parasitol. 42, 1099–1105. doi: 10.1016/j.ijpara.2012.08.007 23022617

[B16] Crans D. C. Meade T. J. (2013). Preface for the fórum on metals on medicine and health: New opportunities and approaches to improving health. Inorg. Chem. 52 (21), 12181–12183. doi: 10.1021/ic402341n 24187925

[B17] Cui L. Su X. Z. (2009). Discovery, mechanisms of action and combination therapy of artemisinin. Expert Rev. Anti Infect. Ther. 7 (8), 999–1013. doi: 10.1586/eri.09.68 19803708 PMC2778258

[B18] Dinio T. Gorka A. Mc Ginniss A. Roepe PD. Morgan J. B. (2012). Investigating the activity of quinine analogues versus chloroquine resistant *Plasmodium falciparum* . Bioorg. Med. Chem. 20 (10), 3292–3297. doi: 10.1016/j.bmc.2012.03.04 22512909 PMC3345081

[B19] Dondorp A. M. Nosten F. Yi P. Das D. Phyo A. P. Tarning J. . (2009). Artemisinin resistance in *Plasmodium falciparum* malaria. N Engl. J. Med. 361, 455e467. doi: 10.1056/NEJMoa0808859 19641202 PMC3495232

[B20] Fabbri C. Trindade A. O. Andrade F. S. Souza M. F. Rios-Velásquez C. M. Lacerda M. V. G. . (2021). Transmission-blocking compound against *Plasmodium vivax* using *P. berghei* as an initial screening. Mem. Inst. Oswaldo Cruz 116, e200513. doi: 10.1590/0074-02760200513 33566952 PMC7874845

[B21] Fields R. D. Lancaster M. V. (1993). Dual attribute continuous monitoring of cell proliferation/cytotoxicity. Am. Biotechnol. Lab. 11 (4), 48–50. 7763491

[B22] Garcia-Bustos J. F. Gamo F. J. (2013). Antimalarial drug resistance and early drug discovery. Curr. Pharm. Des. 19 (22), 270–281. doi: 10.2174/138161213804070357 22973885

[B23] Gardiner D. L. Skinner-Adams T. S. Brown C. L. Andrews K. T. Stack C. M. McCarthy J. S. . (2009). *Plasmodium falciparum*: new molecular targets with potential for antimalarial drug development. Expert Rev. Anti Infect. Ther. 7 (9), 1087–1098. doi: 10.1586/eri.09.93 19883329

[B24] Gokhale N. H. Padhye S. B. Billington D. C. Rathbone D. L. Croft S. L. Kendrick H. D. . (2003). Synthesis and characterization of copper(II) complexes of pyridine-2-carboxamidrazones as potent antimalarial agents. Inorg. Chim. Acta 349, 23–29. doi: 10.1016/S0020-1693-(03)00047-1

[B25] Gokhale N. H. Shirisha K. Padhye B. P. Croft SL. Kendrick H. D. Mckee V. (2006). Metalloantimalarials: Synthesis, X-ray crystal structureof potent antimalarial copper (II) complex of arylazo-4-hydroxy-1,2-naphthoquinone. Bioorg. Med. Chem. Lett. 16 (2), 430–432. doi: 10.1015/j.bmcl.2005.09.061 16275074

[B26] Hofheinz W. Merkli B. (1984). “Quinine and quinine analogues,” in Antimalarial drugs II. handbook of experimental pharmacology, Eds. Peters W. Richards W. H. G. (Berlin, Heidelberg: Springer-Verlag) 68, 61–81. doi: 10.1007/978-3-642-69254-3_2

[B27] Hurwitz E. (1981). Resistance of *Plasmodium falciparum* malaria to sulfadoxine-pyrimethamine (‘Fansidar’) in a refugee camp in Thailand. Lancet 317, 1068–1070. doi: 10.1016/s0140-6736(81)92239-x 6112445

[B28] Krettli A. U. Andrade-Neto V. F. Brandão M. G. L. Ferrari V. M. S. (2001). The search for new antimalarial drugs from plants used to treat fever and malaria or plants randomly selected: a review. Mem. Ins. Oswaldo Cruz 96 (8), 1033–1042. doi: 10.1590/S0074-02762001000800002 11784919

[B29] Krungkrai S. R. Yutharvong Y. (1987). The antimalarial action on plasmodium falciparum of qinghaosu and artesunate in combination with agents which modulate oxidant stress. Trans. R Soc. Trop. Med. Hyg. 5 (81), 710–714. doi: 10.1016/0035-9203(87)90003-4 3329778

[B30] Lacerda M. V. Mourão M. P. Alexandre M. A. Siqueira A. M. Magalhães B. M. Martinez-Espinosa F. E. . (2012). Understanding the clinical spectrum of complicated *Plasmodium vivax* malaria: a systematic review on the contributions of the Brazilian literature. Malar. J. 11, 12. doi: 10.1186/1475-2875-11-12 22230294 PMC3268102

[B31] Lambros C. Vanderberg J. P. (1979). Synchronization of *Plasmodium falciparum* erythrocytic stages in culture. J. Parasitol. 65 (3), 418–420. doi: 10.2307/3280287 383936

[B32] Lu F. Culleton R. Zhang M. Ramaprasad A. von Seidlein L. Zhou H. . (2017). Emergence of indigenous artemisininresistant *Plasmodium falciparum* in Africa. N Engl. J. Med. 376, 991–993. doi: 10.1056/NEJMc1612765 28225668

[B33] Medici S. Peana M. Nurchi V. M. Lachowicz J. I. Crisponi G. Zoroddu M. A. (2015). Noble metals in medicine: latest advances. Coord. Chem. Rev. 284, 329–350. doi: 10.1016/j.ccr.2014.08.002

[B34] Meneguetti D. U. O. Cunha R. M. Lima R. A. Oliveira F. A. S. Medeiros D. S. Passarini G. M. . (2014). Antimalarial ethnopharmacology in the Brazilian Amazon. Rev. Ciênc Farm Basica Apl. 35 (4), 577–587.

[B35] Mohapatra S. C. Tiwari H. K. Singla M. Rathi B. Sharma A. Mahiya K. . (2010). Antimalarial evaluation of copper(II) nanohybrid solids: inhibition of plasmepsin II, a hemoglobin-degrading malarial aspartic protease from *Plasmodium falciparum* . J. Biol. Inorg. Chem. 15 (3), 373–385. doi: 10.1007/s00775-009-0610-9 19946719

[B36] Moore D. V. Lanier J. E. (1961). Observations on two *Plasmodium falciparum* infections with an abnormal response to chloroquine. Am. J. Trop. Med. Hyg. 10, 5–9. doi: 10.4269/ajtmh.1961.10.5 13772281

[B37] Murambiwaa P. Masolab B. Govenderc T. Mukaratirwad S. Musabayanea C. T. (2011). Anti-malarial drug formulations and novel delivery systems: A review. Acta Trop. 118 (2), 71–79. doi: 10.1016/j.actatropica.2011.03.005 21439929

[B38] Na-Bangchang K. Karbwang J. (2013). Emerging artemisinin resistance in the border areas of Thailand. Expert Rev. Clin. Pharmacol. 6 (3), 307–322. doi: 10.1586/ecp.13.17 23656342

[B39] Navarro M. Gabiani C. Messori L. Gambino D. (2010). Metal-based drugs for malaria, trypanosomiasis and leishmaniasis: recent achievements and perspectives. Drug Discovery Today 15 (23-24), 1070–1078. doi: 10.1016/j.drudis.2010.10.005 20974285

[B40] Noedl H. Se Y. Schaecher K. Smith B. L. Socheat D. Fukuda M. M. (2008). Evidence of artemisinin-resistant malaria in western Cambodia. N Engl. J. Med. 359 (24), 2619–2620. doi: 10.1056/NEJMc0805011 19064625

[B41] Obaleye J. A. Tella A. C. Arise R. O. (2009). *In vivo* antimalarial activity and toxicological studies of some quinoline methanol metal complexes. Adv. Nat. Appl. Sci. 3 (1), 43–48.

[B42] OECD . (2022). Test no. 425: Acute oral toxicity – up-and-down procedure, OECD guidelines for the testing of chemicals, section 4 (Paris: OECD Publishing). doi: 10.1787/9789264071049-en

[B43] Olliaro P. (2001). Mode of action and mechanisms of resistance for antimalarial drugs. Pharmacol. Ther. 89 (2), 207–219. doi: 10.1016/s0163-7258(00)00115-7 11316521

[B44] Orvig C. Abrams M. J. (1999). Medicinal inorganic chemistry: introduction. Chem. Rev. 99 (9), 2201–2204. doi: 10.1021/cr980419w 11749478

[B45] Payne D. (1987). Spread of chloroquine resistance in *Plasmodium falciparum* . Parasitol. Today 3, 241–246. doi: 10.1016/0169-4758(87)90147-5 15462966

[B46] Raposo C. C. B. S. Santos J. B. Santos G. M. C. Gonçalves E. G. R. Silva A. R. (2013). *Plasmodium vivax* malaria: related factors to severity in the state of maranhão. Rev. Soc. Bras. Med. Trop. 46 (1), 67–72. doi: 10.1590/0037-868212382013 23563828

[B47] Rios-Velásquez C. M. Martins-Campos K. M. Simões R. C. Izzo T. dos Santos E. V. Pessoa F. A. C. . (2013). Experimental *Plasmodium vivax* infection of key *Anopheles* species from the Brazilian Amazon. Malar. J. 12, 460–470. doi: 10.1186/1475-2875-12-460 PMC3878095 24359307

[B48] Salas P. F. Herrmann C. Orvig C. (2013). Metalloantimalarials. Chem. Rev. 113 (5), 3450–3492. doi: 10.1021/cr3001252 23425067

[B49] Sanchez-Delgado R. A. Anzellotti A. (2004). Metal complexes as chemotherapeutic agents against tropical diseases: trypanosomiasis, malaria and leishmaniasis. Mini Rev. Med. Chem. 4 (1), 23–30. doi: 10.2174/1389557043487493 14754440

[B50] Sekhon B. S. Bimal N. J. (2012). Transition metal-based antimalarial. Am. J. Pharm. Educ. Res. 3 (2), 52–63.

[B51] Stanila A. Braicu C. Stanila S. Pop R. M. (2011). Antibacterial activity of copper and cobalt amino acids complexes. Not. Bot. Horti. Agrobot. Cluj-Na. 39 (2), 124–129. doi: 10.15835/nbha3926847

[B52] Subczynski W. K. Antholine W. E. Hyde J. S. Petering D. H. (1987). Orientation and mobility of a copper-planar complex in a lipid bilayer. J. Am. Chem. Soc 109 (1), 46–52. doi: 10.1021/ja00235a007

[B53] Tapanelli S. Habluetzel A. Pellei M. Marchió L. Tombesi A. Capparè A. . (2017). Novel metalloantimalarials: Transmission blocking effects of water soluble Cu(I), Ag(I) and Au(I) phosphane complexes on the murine malaria parasite *Plasmodium berghei* . J. Inorg. Biochem. 166, 1–4. doi: 10.1016/j.jinorgbio.2016.10.004 27815977

[B54] Trager W. Jensen J. B. (1976). Human malaria parasites in continuous culture. Science 193 (4254), 673–675. doi: 10.1125/science.781840 781840

[B55] Valecha N. Phyo A. P. Mayxay M. Newton P. N. Krudsood S. Keomany S. . (2010). An open-label, randomised study of dihydroartemisinin-piperaquine versus artesunate-mefloquine for falciparum malaria in Asia. PloS One 5 (7), e11880. doi: 10.1371/journal.pone.0011880 20689583 PMC2912766

[B56] Vanaerschot M. Lucantoni L. Li T. Combrinck J. M. Ruecker A. Kumar T. R. S. . (2017). Hexahydroquinolines are antimalarial candidates with potent blood stage and transmission-blocking. Nat. Microbiol. 2 (10), 1403–1414. doi: 10.1038/s41564-0147-0007-4 28808258 PMC5708124

[B57] Wadi I. Nath M. Anvikar A. R. Singh P. Sinha A. (2019). Recent advances in transmission-blocking drugs for malaria elimination. Future Med. Chem. 11 (23), 3047–3089. doi: 10.4155/fmc-2019-0225 31782936

[B58] Warhurst D. C. (1981). Cinchona alkaloids and malaria. Lancet 12 (8259), 1346. doi: 10.1016/s0140-6736(81)91364-7 6118739

[B59] Weintraub S. Moskovitz Y. Fleker O. Levy A. R. Meir A. Ruthstein S. . (2015). SOD mimetic activity and antiproliferative properties of a novel tetra nuclear copper (II) complex. J. Inorg. Chem. 20 (8), 1287–1298. doi: 10.1007/s00775-015-1307-x 26547749

[B60] Weselucha-Birczynska A. (2004). Aggregation phenomena of cinchonine in aqueous solutions observed and analysed by 2D FT-raman spectroscopy. Vib. Spectrosc. 35 (1-2), 189–198. doi: 10.1016/j.vibspec.2004.01.011

[B61] Weselucha-Birczynska A. Oleksyn B. J. Hoffmann S. K. Śliwinski J. Borzecka-Prokop B. Goslar J. . (2001). Flexibility of CuCl4-tetrahedra in bis[cinchoninium tetrachlorocuprate(II)] trihydrate single crystals. X-ray diffraction and EPR studies. Inorg. Chem. 40 (18), 4526–4533. doi: 10.1021/ic001402a 11511195

[B62] White N. Qiao L. G. Qi G. Luzzatto L. (2012). Rationale for recommending a lower dose of primaquine as a *Plasmodium falciparum* gametocytocide in populations where G6PD deficiency is common. Malar. J. 11, 418. doi: 10.1186/1475-11-418 23237606 PMC3546849

[B63] World Health Organization (2018) Policy brief on single-dose primaquine as a gametocytocide in plasmodium falciparum malaria. Available at: www.who.int/malaria/publications/atoz/policy-brief%20-single-dose-primaquine-pf/en/.

[B64] World Health Organization (2021). World malaria report (Geneva: WHO publications). Licence: CC BY-NC-SA 3.0 IGO. ISBN 978-92-4-004049-6.

[B65] Yeung S. Socheat D. Moorthy V. S. Mills A. J. (2009). Artemisinin resistance on the Thai-Cambodian border. Lancet 374 (9699), 1417–1419. doi: 10.1016/S0140-6736(09)61856-0 19854365

